# New products

**DOI:** 10.1155/S1463924693000124

**Published:** 1993


Journal of Automatic Chemistry, Vol. 15, No. 2 (March–April 1993), pp. 73–77

### New products

### Automated SPE technology for water analysis

The AutoTrace SPE Workstation automates the time-consuming and often error-prone steps in Solid Phase Extraction (SPE) of trace contaminants from water. Through the use of precision syringes, pumps, and easy-to-use menu software, the AutoTrace processes up to six water samples simultaneously. Sample volumes can range from as low as 10 l to as high as 2 l; and five reagents can be used for conditioning and elution steps. The AutoTrace uses 1, 3, 6 or 8 ml syringe barrel SPE cartridges, which can be dried with an inert gas if desired. The eluent receiving containers can be standard 16X100 test-tubes, WISP or GC vials. The waste stream separates the water and organics for easier and less costly solvent disposal.

The conditioning, sample delivery and elution steps are microprocessor-controlled, which improves the precision of the results. The unit runs with its own internal operating system no dedicated computer is necessary. The methods are developed on a personal computer using pull-down menus. The software easily accomodates multiple elution steps from a single SPE cartridge with minimal manual intervention to change the receiving tubes.

*Details from Sharon Correia, Zymark Corporation, Hopkinton, Massachusetts 01748, USA. Tel.: 508 435 9500; fax: 508 435 3439.*

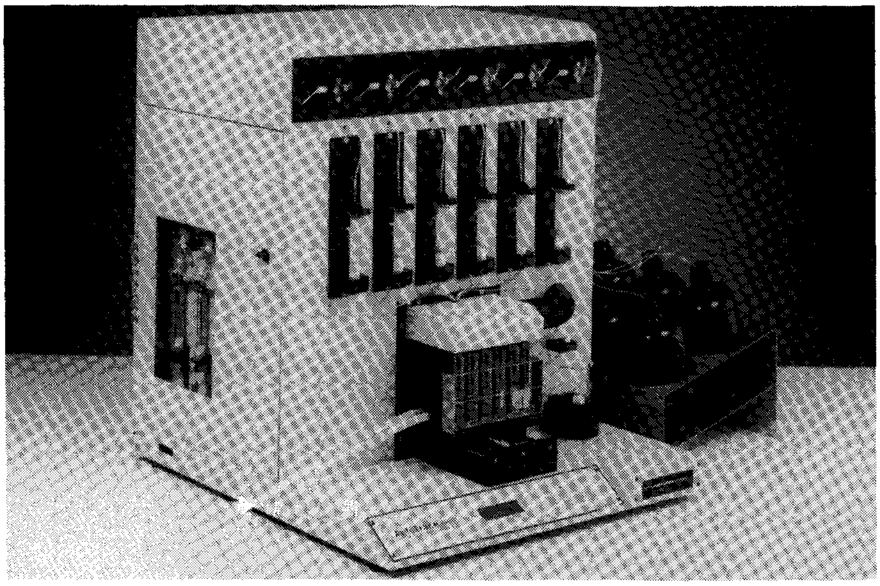

*Zymark's AutoTrace SPE Workstation, which can process over 24, one litre water samples in an eight hour shift and requires only 15 minutes of operator time (see text).*

### Networking software

Hewlett-Packard's HP ChemLAN (DOS Series) networking software is designed to link HP ChemStation (DOS Series) applications (gas chromatography, mass spectrometry and liquid chromatography) on a standards-based network. This allows R&D, QC, QA and other laboratories with a high throughput of samples to increase data analysis capabilities by using client/server computing.

HP ChemLAN (DOS Series) software offers a number of advantages, for example: automatic DOS-to-DOS HP ChemLAN sequencing; automatic DOS-to-DOS printing, including graphics; transparent remote network file system (NFS) access to HP-UX* systems; and simultaneous client/server functionality.The automatic DOS-to-DOS HP ChemLAN sequencing function of the HP ChemLAN (DOS Series) server software reduces both analysis and reporting time and allows laboratories to increase throughput. When data acquisition is completed on one HP ChemStation, the data, along with the sequence, are sent automatically to another HP ChemStation on the network, where analysis and reporting are performed without operator intervention. The data acquisition ChemStation continues to collect new data while the reporting ChemStation processes it. Multiple HP ChemStations across the laboratory can acquire data for automatic transfer to a single reporting ChemStation.

HP ChemLAN (DOS Series) server software allows automated, remote DOS-to-DOS printing, eliminating the need for a separate printer at each workstation and providing the whole laboratory with access to high-quality printing. Reports including graphics such as chromatogram and spectra, word processed documents and spreadsheets can all be printed from HP ChemStations or any other DOS-based system running HP ChemLAN (DOS Series) client software and Microsoft Windows. Unlike many print servers, the server running HP ChemLAN (DOS Series) need not be dedicated to printing.

HP ChemLAN (DOS Series) software also give laboratories transparent access to remote disks through NFS functionality, making it easy for systems to share method, data and sequence files while maintaining file security. Laboratories can access files on an HP-UX ChemSystem just as if they were on the local system. NFS also provides password protection.

A new feature of HP ChemLAN (DOS Series) software is its ability to run client and server network programs simultaneously, resulting in significant gains in laboratory productivity. HP ChemLAN users can now send and receive information at the same time; for example, they can receive data over the laboratory network while sending a final report

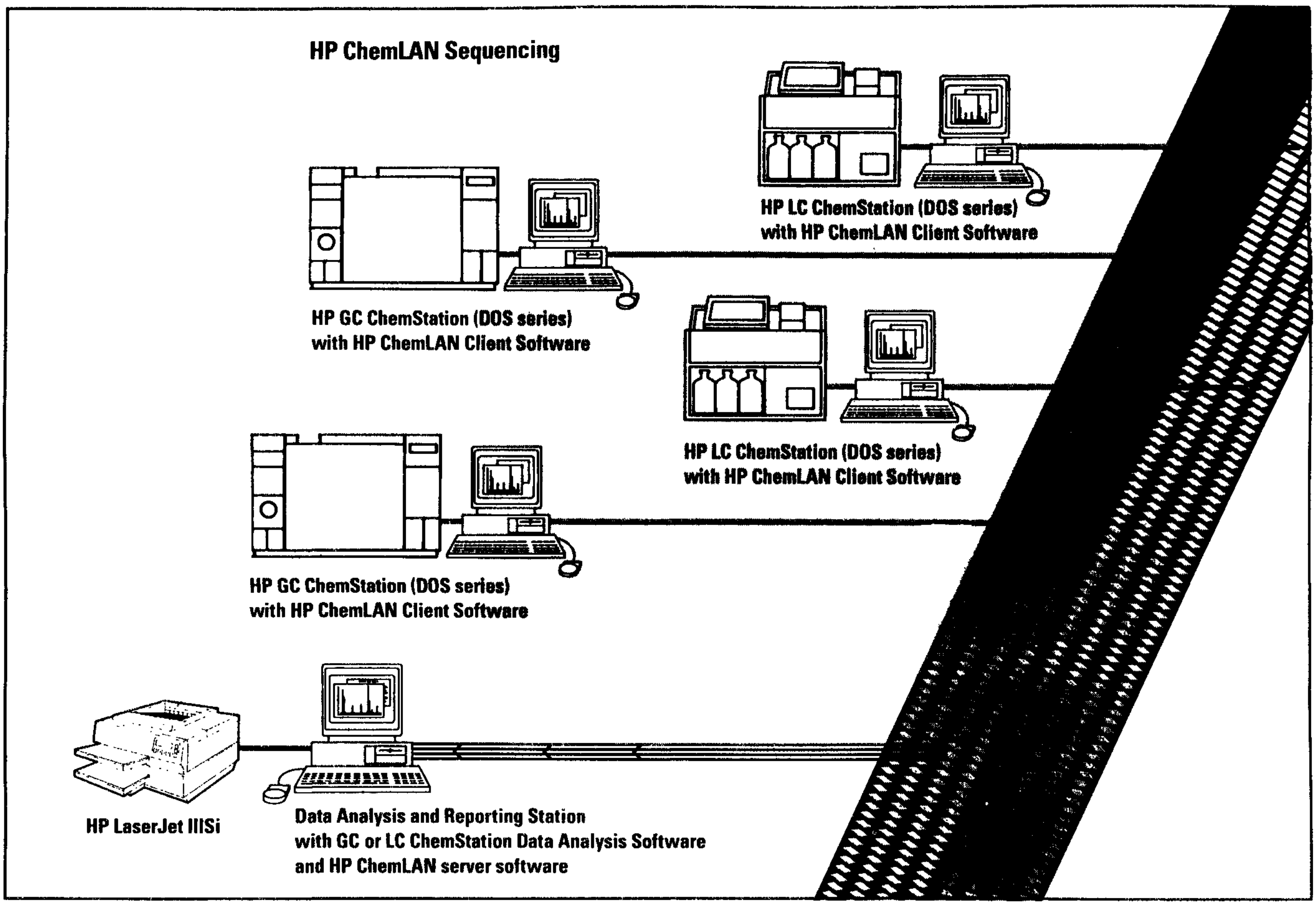

*HP ChemLAN sequencing is the co-ordinated execution of data acquisition on client systems and data analysis and reporting on server systems (see text).*

to the Laboratory Information Management System (LIMS).

*Enquiries to Verena Haller, Hewlett-Packard SA, 150 Route du Nant-d'Avril, CH-1217 Meyrin (GE) 2, Switzerland.*

### Uninteruptable power systems

The Galatrek 500VA to 3kVA MicroBak Uninterruptible Power Systems (UPS) offer new communication and control facilities for critical PCs and file servers. Features include a front-panel LCD display with scroll up and down chevrons, ensuring an easy to use source of critical operational information. Previously, this could only be guessed at from front panel LEDs and a single audible tone alarm.

Any of the 20 different meter functions available can be shown including battery charge %, number of minutes remaining and the actual load in VA and W. The LCD display is also used by the UPS to communicate site for functional problems and warning messages are shown on the display.

Each MicroBak is designed and manufactured in the UK and also incorporates a morse code audible alarm and an RS-232 port. The UPS can used this serial link to communicate with a standard terminal, the MicroBak hand held keypad, a palmtop computer or any computer running PowerCheck Plus software.

*Further information from Galatrek International Ltd, Scotland Street, Llanrwst, Gwynedd LL26 0AL, UK. Tel.: 0492 640311.*

### EDX detectors

Oxford Instruments Microanalysis Division has launched the Pentafet Plus range of detectors. Already combining the highest count rate capability and resolution sensitivity available, preformance is now improved in three important areas. A Super-ATW window gives Be detection with a sealed detector; it offers an improved resolution specification with the added convenience of thermal cycling (the detector can be allowed to warm up when not in use).

Super-ATW is a sealed detector which combines the unique advantages of Pentafet detector technology, with the best transmission available in thin film window technology, Backed by guaranteed resolutions of 75 eV at fluorine, 133 eV at manganese at 1000 Cps plus the unrivalled resolution of 148 eV at manganese at 10 000 Cps acquisition rate, the detector exceeds the traditional IEEE specification of guaranteeing resolution from an Fe55 source on the bench.

*Enquiries to Janet Wood at Oxford Instruments (UK) Ltd, Microanalysis Group. Halifax Road, High Wycombe, Buckinghamshire HP12 3SE, UK. Tel.: 0494 442255.*

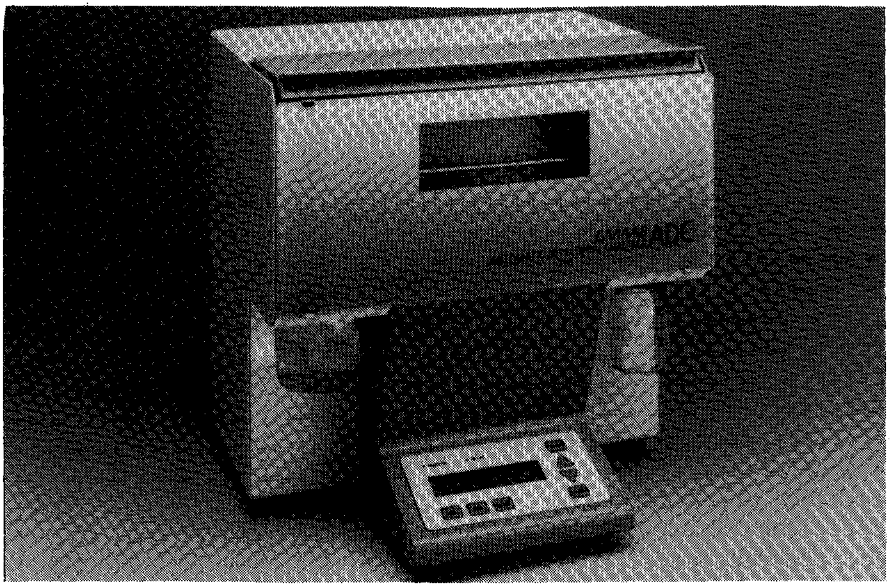

*CAMAG's Automatic Devloping Chamber (ADC) for Thin-Layer Chromatography offers complete control of the developing conditions from plate to plate. Operation is simple: parameters are selected, for example tank or sandwich configuration, running distance and plate drying conditions. Developing programs can be stored for easy recall and use. Details from CAMAG, Sonnenmattstrasse II, CH 4132 Muttenz, Switzerland.*

### Melting point apparatus

The Automelt IA2001, a fully automatic melting-point apparatus can accurately and simultaneously analyse up to three disparate samples.

The basis of the instrument's operating procedure is an electro-optical technique. This enables the instrument to thermally analyse a wide spectrum of sample materials over a temperature range from ambient to +400 °C. The Automelt has a menu-driven user interface; in a typical determination, samples (in standard melting-point tubes) are heated through their melting-point at any pre-selected ramp rate from 0·2 °C to 10 °C/min in 0·1° steps. Following computer processing, the characteristics of the melts are shown graphically on the integral LCD display. Start and final melt temperatures are automatically recorded with an accuracy of ±0·2 °C.

Although designed to be used without operator intervention, there is a manual override facility. With this, the user can designate any additional critical points of visual interest with manually-added marker flags. Melts are observed through the Automelt's 20× stereo binocular viewing head.

Displayed figure can be expanded and manipulated to show excellent detail; and all results will be stored in memory for later analysis. Memory data can be accessed and displayed at any time, allowing easy comparison with reference sample(s). Furthermore, data can be recorded with optional codes: types of materials being analysed, operator or batch numbers, as required.

Integral floppy disk storage is provided as standard, and there is a printer output port.

*Details from Electrothermal Engineering Ltd, 419 Sutton Road, Southend-on-Sea, Essex SS2 5PH, UK. Tel.: 0702 612211; fax: 0702 619888.*

### Automated random testing

Any recording system for testing of human samples in laboratories should be 100% accurate. Elimination of human error in recording data through the use of bar-code technology has been an important breakthrough for test laboratories.

Computype has announced a bar-code to be attached to cuvettes for batch testing providing instant, accurate and automatic recording and monitoring of all data. The bar-code labels feature high-print contrast and durability and are guaranteed to be read at first scan. In addition, they are numbered sequentially so that each label

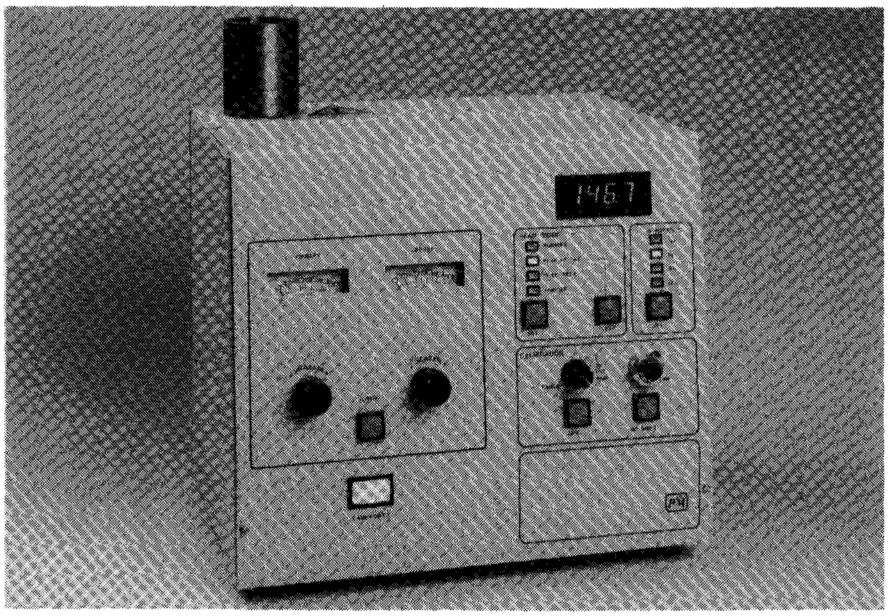

*P S Analytical has improved the sensitivity of the Excalibur atomic fluorescence detector for the determination of As, Se, Sb and Te. This significant advance has been achieved by the use of multi-reflectance filters to improve the light transmission. The use of these filters has resulted in a 10-fold improvement, giving a detection limit for As of 10 parts per trillion. Contact P S Analytical Ltd, Arthur House, B4 Chaucer Business Park, Watery Lane, Kemsing, Sevenoaks, Kent TN15 6QY, UK. Tel: 0732 763416; Fax 0732 761340.*

is unique, giving an individual identity to each cuvette to which it is attached.

The labels are small (1 cm deep and 4 cm wide) and carry eye-readable information, as well as the bar-code. In addition, a special adhesive has been applied to ensure the labels remain in place throughout testing.

Computype's bar-codes exceed the specification laid down by the major European scanner manufacturers and are produced to European quality standard ISO 9002 (BS 5750).

*77Further information from Computype Ltd, COMPU/140, PO Box 6, Clifton, Nottingham NG11 6PW, UK.*

### Microsampling and automatic sample pretreatment

An intelligent sampling station with the Shimadzu AA6501 series Atomic Absorption Spectrophotometer, sold through V. A. Howe & Company Ltd, performs automatic sample pretreatment using up to six reagents. In addition, a single stock standard can be diluted to produce a calibration curve; over range samples are diluted; and 60 samples can be remeasured. The sampling station can be used for flame analysis (as low as 100 μl volume) and graphite furnace analysis.

*For further information contact V. A. Howe & Company Ltd, Beaumont Close, Banbury, Oxfordshire OX16 7RG, UK. Tel.: 0295 252666; fax: 0295 268096.*

### Biokinetic accesory for Perkin-Elmer Model LS-50B

The new computer-controlled biokinetic accessory for the Perkin-Elmer Model LS-50B allows researchers to regulate the temperature and the stirring speed of cell samples. It consists of a single-position thermostatted cell holder which accommodates a 10 mm cuvette, whose contents can be stirred at a speed set by the LS-50B integral software. A temperature probe monitors the block temperature and can be calibrated to report the temperature of the sample in the cuvette. Fluorescence intensity and temperature data are available from the software.

The biokinetic accessory allows the user to mark the addition of each new substance to the cuvette. A separate time file is generated, containing all marked events.

*For further information contact Perkin-Elmer Ltd, Post Office Lane, Beaconsfield, Buckinghamshire HP9 1QA, UK. Tel.: 0494 679026; fax: 0494 679064.*

### Continuous ion-exchange gas dryers

P S Analytical has recently introduced a continuous gas drying system utilizing a hygroscopic ion-exchange membrane. This unit, called the 'Perma-Pure' dryer, is ideally suited for such techniques as AA, ICP, DCP, atomic fluorescence and purge and trap GC, where increased levels of moisture in the gas stream will reduce the performance of the system.

*For further information contact P S Analytical Ltd, Arthur House, B4 Chaucer Business Park, Watery Lane, Kemsing, Sevenoaks, Kent TN15 6QY, UK. Tel.: 0732 63416; fax: 0732 61340.*

### Cell Robotics, Inc.

Cell Robotics, Inc. have announced two new products: the LaserTweezers 1000 system for optical trapping and micromanipulation and the FT 1000 Fourier transform spectral imaging system.

The LaserTweezers 1000 converts a standard inverted microscope into a laser-based optical trapping system. This system can then be used to trap, levitate and manipulate single cells and other microscopic particles (for example chromosomes). An infra-red diode laser is used as the trapping beam, so that these manipulations are performed without physical contact or damage to the cell.

The LaserTweezers is useful in a wide varity of research applications. Rare cells, chromosomes or other microscopic particles can be separated with absolute purity. Selected cells can be placed in contact with one another so as to analyse their interactions. These and other operations are performed entirely within Cell Robotics' proprietary cell manipulation chamber.

The FT 1000 spectral imaging system represents a new generation of fluorescence microscopes. Its primary function is to image the spatial distribution of large numbers of spectral parameters and fluorescent probes. It does so without using optical filters or beam splitters, resulting in minimum light loss and a greatly simplified optical layout. This is an instrument that can be used for a multitude of biological applications in which it is important to identify and/or characterize certain types of cells and cellular structures that have been labelled with fluorescent probes.

*Details from Cell Robotics, Inc., 2715 Broadbent Parkway NE, Albuquerque, New Mexico 87107, USA.*

### Differential thermal analyser

Perkin-Elmer's DTA 7 Differential Thermal Analyser permits direct temperature and energy measurement by DTA, or heat flux DSC techniques, of the endothermic and exothermic behaviour of a wide variety of samples at temperatures ranging between ambient and 1600 °C.

Fully computer-controlled, with a range of powerful standard and optional software, the DTA 7 is the latest addition to the multitasking, multi-analyser 7 Series/UNIX Thermal Analysis System. It can perform simultaneous, independent analyses with the high temperature TGA 7 Thermogravimetric Analyser for any other Perkin-Elmer 7 Series Thermal Analyser. The compact design facilitates convenient siting in the laboratory.

The newly-designed furnace is kinetically mounted to ensure repeatable positioning and high base-line reproducibility, allowing accurate measurement of specific heat values. Rapid cooling provided by an automatic force-air cooling system improves sample throughput and increases productivity. The DTA 7 will be of particular interest to analysts working in the minerals, ceramic, inorganic materials, paper, rubber, paint and glass industries.

*For further information, contact Perkin-Elmer Ltd, Post Office Lane, Beaconsfield, Buckinghamshire HP9 1QA, UK. Tel.: 0494 679026; fax: 0494 679064.*

### Colorimetric analysis software

COLOR V1.0G is Windows-based software that performs colorimetric analysis on spectral data. It executes a variety of correction factors including base-line, dark reference, and absolute whiteness. Calculations supported include tristimulous values, chromaticity, CIE L*A*B, CIE L*U*V, hunter, metric distance, whiteness, and yellowness. Calculations can be performed over standard wavelength ranges or over a range specified by the user. The software can analyse data singly from sorted data files, from a batch list of data, or directly from spectrophotometers supported by Galactic Data Acquisition software.

COLOR runs under Galactic's GRAMS/386 software on 386 and 486 PCs and compatibles. It requires Windows V3.0, a math co-processor, and VGA display.

*For additional information contact Kelly W. McIntire, Galactic Industries Corporation, 395 Main Street, Salem, New Hampshire 03079, USA. Tel.: 603 898 7600; fax: 603 898 6228.*

### System suitability testing

An application package, called 'SysSuit' is available for Perkin-Elmer Model 1020 Integrators and the 1020 LC Plus Controller. The package tests the performance of the entire chromatographic system, including the column, injector, detector, electronics, mobile phase, operating temperature and other components.

SysSuit verifies that the system is operating within the performance requirements of an analytical method and provides comparisons between different systems. Suitability testing is used periodically between samples as a routine quality-control measure and when a system component is replaced.

The system is evaluated for separation characteristics against a series of parameters with user-defined limits. If the parameters fall within these limits, the system is deemed 'suitable'. Values for the parameters are calculated according to USP (United States Pharmacopoeia) or BP (British Pharmacopoeia) guidlines to meet regulatory requirements.

*Further information from Perkin-Elmer Ltd, Post Office Lane, Beaconsfield, Buckinghamshire HP9 1QA, UK. Tel.: 0494 679026; fax: 0494 679064.*

